# Neurogenic substance P—influences on action potential production in afferent neurons of the kidney?

**DOI:** 10.1007/s00424-021-02552-z

**Published:** 2021-03-30

**Authors:** Kristina Rodionova, Karl F. Hilgers, Peter Linz, Johannes Schätzl, Giulia Raschke, Christian Ott, Roland E. Schmieder, Mario Schiffer, Kerstin Amann, Roland Veelken, Tilmann Ditting

**Affiliations:** 1 Nephrological Laboratories, Department of Internal Medicine 4 – Nephrology and Hypertension, Friedrich-Alexander University Erlangen, Loschgestrasse 8, 91054 Erlangen, Germany; 2 Department of Radiology, Friedrich-Alexander University Erlangen, Erlangen, Germany; 3 Department of Internal Medicine 4 – Nephrology and Hypertension, Paracelsus Private Medical School Nuremberg, Nuremberg, Germany; 4 Department of Nephropathology, Friedrich-Alexander University Erlangen, Erlangen, Germany

**Keywords:** SP, CGRP, Renal innervation, Neuronal cell culture, Electrophysiology, DRG

## Abstract

We recently showed that a substance P (SP)–dependent sympatho-inhibitory mechanism via afferent renal nerves is impaired in mesangioproliferative nephritis. Therefore, we tested the hypothesis that SP released from renal afferents inhibits the action potential (AP) production in their dorsal root ganglion (DRG) neurons. Cultured DRG neurons (Th11-L2) were investigated in current clamp mode to assess AP generation during both TRPV1 stimulation by protons (pH 6) and current injections with and without exposure to SP (0.5 µmol) or CGRP (0.5 µmol). Neurons were classified as tonic (sustained AP generation) or phasic (≤ 4 APs) upon current injection; voltage clamp experiments were performed for the investigation of TRPV1-mediated inward currents due to proton stimulation. Superfusion of renal neurons with protons and SP *increased* the number of action potentials in tonic neurons (9.6 ± 5 APs/10 s vs. 16.9 ± 6.1 APs/10 s, *P* < 0.05, mean ± SD, *n* = 7), while current injections with SP *decreased it* (15.2 ± 6 APs/600 ms vs. 10.2 ± 8 APs/600 ms, *P* < 0.05, mean ± SD, *n* = 29). Addition of SP significantly *reduced* acid-induced TRPV1-mediated currents in renal tonic neurons (− 518 ± 743 pA due to pH 6 superfusion vs. − 82 ± 50 pA due to pH 6 with SP superfusion). In conclusion, SP increased action potential production via a TRPV1-dependent mechanism in acid-sensitive renal neurons. On the other hand, current injection in the presence of SP led to decreased action potential production. Thus, the peptide SP modulates signaling pathways in renal neurons in an unexpected manner leading to both stimulation and inhibition of renal neuronal activity in different (e.g., acidic) environmental contexts.

**Supplementary Information:**

The online version contains supplementary material available at 10.1007/s00424-021-02552-z.

## Introduction

Although the publication of results of the SYMPLICITY 3 trial in 2015 [3] almost put an end to investigation of renal nerve ablation for the treatment of high blood pressure, recent studies with new technical approaches [1, 5, 21, 56] led to smaller blood pressure decreases than the original studies but suggest a further role of renal nerve ablation in the therapy of arterial hypertension [42].

The exact mechanisms by which the denervation of a single sympathetically innervated area, e.g., the kidney, actually leads to a decrease in arterial blood pressure with lower sympathetic central outflow are still not fully understood. The main effect of the renal sympathetic system influences salt and water excretion [12]; however, so far no lasting physiological changes could be observed in hypertensive patients after renal denervation, which could explain blood pressure decreases after renal nerve ablation [43].

In this context, the afferent innervation to the kidney repeatedly came into focus [11, 12]. Even clinical studies suggest a role of afferent renal nerves in controlling the sympathetic nervous system [10, 26]. While those reports suggest that afferent renal nerve traffic influences efferent sympathetic activity [2, 11, 30, 31], many investigations have also demonstrated that afferent nerve fibers release their main neuropeptides substance P (SP) and calcitonin gene–related peptide (CGRP) influencing local circulation and immune responses [9, 17, 58].

SP-positive nerve fibers can be found in the kidney close to afferent and efferent arterioles and in the glomeruli. Since macrophages and dendritic cells were detected in close vicinity to SP containing sensory neurons in the kidney, a role in neuroimmunomodulation is very likely [46, 57]. CGRP is a potent vasodilator released from peptidergic afferent nerve fibers, thus contributing to blood flow regulation [6, 45, 52] that will likely interfere with immune responses.

We could recently demonstrate that a SP-dependent sympatho-inhibitory mechanism [14] linked to afferent renal innervation is impaired in normotensive mesangioproliferative nephritis in rats, while at the same time neuronally released SP augments intrarenal inflammation [48].

The question now arises whether SP released in the kidney by dendrites of afferent nerves also has an effect on the neurons themselves from which it is released, thereby influencing neurophysiological phenomena such as action potential (AP) production.

Afferent renal nerve fibers are difficult to investigate in vivo. Therefore, we developed a primary neuronal cell culture model allowing to investigate dorsal root ganglion neurons with renal afferent nerve fibers in vitro [16, 23]. We could demonstrate previously that the number of tonic renal neurons, characterized by sustained AP firing during current injection, was significantly decreased if the neurons were incubated with the inflammatory cytokine CXCL1 [13]. Based on these observations, we aimed to test the hypothesis that substance P impairs the action potential production of neurons with projections from the kidney. We chose two ways to stimulate cultured DRG neurons to produce action potentials: Firstly, we used the direct administration of protons (pH 6) onto cultured neurons to stimulate TRPV1 receptors [16].

In a second experimental approach, current injections [13, 16] were applied.

## Materials and Methods

Sprague–Dawley rats (Charles River, Kisslegg, Germany) weighing 180–250 g (9 to 12 weeks of age) were maintained in our animal facility at 24 ± 2 °C. They were fed a standard rat diet (no. C-1000, Altromin, Lage, Germany) containing 0.2% sodium by weight and had free access to tap water. All procedures performed in animals were done in accordance with the guidelines of the American Physiological Society and in compliance with NIH Guide for Animal Care and Use in Laboratory Practice.

### Labelling of renal afferent neurons

To identify DRG cells that project to the kidney, dicarbocyanine dye (DiI, 1,1′ dioleyl-3,3,3′ tetramethyl-indocarbocyanine methanesulfonate, in EtOH; Molecular Probes®, Darmstadt, Germany) was applied to both kidneys by subcapsular application (5 μl of a solution of 10 g DiI/l) under isoflurane anesthesia as described previously [23]. Anesthesia was induced by use of a plexiglass induction chamber which was flooded with a mixture of 2–3% isoflurane (Forene®, Abbott AG, Baar, Switzerland), and O_2_ plus N_2_O (1:1, respectively) as the carrier gas (Linde Gas Therapeutics, Unterschleißheim Germany). To this end, a gas mixer of an anesthetic apparatus was used (Trajan 808 equipped with Vapor 19.3; Dräger, Liebefeld, Switzerland). Maintenance of anesthesia in spontaneous breathing animals was obtained after the switch to a custom-made face mask with an exhalation valve to avoid accumulation of CO_2_, while the concentration of isoflurane was reduced to 1.5%.

The lower renal poles were exposed through a small flank incision. Rats were treated subcutaneously with a single dose of meloxicam (1 mg/kg) for periprocedural analgesia [51].

The described technique for staining neurons has been used by us for many years and was described in several publications [13, 15, 16, 36, 37, 51]. It has also been successfully used by others [18, 22, 24, 25]. Using this approach, after renal denervation, it is no longer possible to detect labelled neurons in the dorsal root ganglia Th12 to L2, where the first neurons of renal afferent nerve fibers can be found**.**

### Neuronal cell culture

Six days after the labelling procedure, deeply anesthetized rats were euthanized by exsanguination after removal of the spine segment T11–L2 and DRGs were collected from these segments [51]. Neurons were only cultured from animals in which a routine inspection showed that the renal capsule was tight and that no DiI had penetrated into the neighboring tissue.

Neurons were isolated by mechanical and enzymatic dissociation as described previously [13, 36, 51]. The ganglia were incubated with collagenase IA (2 mg/ml C9891, Sigma-Aldrich, Munich, Germany in DMEM, PAA Laboratories GmbH, Linz, Austria) for 1 h in 5% CO_2_ at 37 °C. Enzymatic dissociation was terminated by replacing collagenase-containing DMEM with fresh DMEM + cultural medium (DMEM + , i.e., DMEM plus 10% FCS, 1% penicillin/streptomycin, and 0.1% insulin). Tissue digestion was stopped by FCS. Ganglia were triturated using sterile Pasteur pipettes (Sigmacote®; Sigma-Aldrich, Munich, Germany) to dissociate individual cells. After centrifugation at 100 rcf, cells were resuspended in 10 ml DMEM + and centrifuged once more. The pellet was resuspended in 1.8 ml DMEM + and cells were plated on glass coverslips coated with poly-L-lysine. The coverslips were cultured in DMEM + for 1 day before electrophysiological experiments.

When the cells of the dorsal root ganglion were dissociated, cells besides neurons were almost exclusively fibroblasts and macrophages that could be easily distinguished from the neuronal cells: Neuronal cells exhibit a characteristic large rounded soma (20–40 µm). Furthermore, fibroblasts and macrophages did not produce action potentials.

### Investigation of cultivated neurons

Recordings were made within 30 h of plating. Patch clamp recordings were obtained using a pipette solution containing 140 mM KCl, 5 mM NaCl, 2 mM MgCl_2_, 1 mM CaCl_2_, 2 mM Mg-ATP, 0.3 mM Na-GTP, 10 mM EGTA, and 10 mM HEPES (pH 7.4). Recordings were conducted in whole-cell mode. Patch pipettes were pulled from borosilicate glass capillaries (GB150F-8P; Science Products, Hofheim, Germany) in a two-stage process using a microelectrode puller and a microforge (PP-830, Narishige, Tokyo, Japan) to adjust the opening diameter and a resistance at 2–4 MΩ.

Patch clamp recordings were obtained with an Axopatch 200B amplifier (Axon Instruments, Foster City, CA). Data were sampled at 5 kHz for voltage and 20 kHz for current clamp and analyzed with pClamp® 10.2 (Axon Instruments, Foster City, CA).

Only neurons with a resting membrane potential below − 40 mV were measured. Cells that stained brightly for DiI to laser excitation (540 nM) were considered as renal afferent neurons. Non-renal neurons from the same cultures that showed no DiI staining at all were also tested [36]. All recordings were done at room temperature, i.e., 22 ± 2 °C.

Cell capacitance was compensated manually and cell parameters (size, capacitance, and resistances) were documented. To confirm vitality of each neuron, typical neuronal currents were examined using a voltage step protocol (step duration of 50 ms, from − 100 mV to + 60 mV in 17 steps with a 1-s delay). Neurons were identified by the presence of fast sodium currents during repolarization.

### Current clamp protocols—Effects of SP and CGRP

To determine DRG firing patterns, we utilized a whole-cell current clamp approach previously described [16, 51, 54]. pClamp® 10.2 (Axon Instruments, Foster City, CA) was used to control current-pulse generation, to record membrane potentials, and for off-line data analysis.

To start patch clamp recordings, coverslips containing DRG neurons were transferred to a laminar flow chamber placed on an inverted phase-contrast microscope (Wilowert, Hund, Germany). The chamber was constantly rinsed with a standard extracellular bath solution containing (in mM) 140 NaCl, 5 KCl, 2 CaCl_2_, 1 MgCl_2_, 10 HEPES, and 10 glucose, with pH adjusted to 7.4 with NaOH, at a flow rate of 0.5–1 ml/min by gravity-fed lines, connected to fluid reservoirs and a computer-controlled perfusion system (AutoMate Scientific, USA). A multi-barrel perfusion pipette with a diameter of 140 µm was positioned 600 μm from a target neuron while the superfusate was delivered from a reservoir with a flow pressure of 20 cm H_2_O.

### Acid stimulation

In one set of experiments, neurons were stimulated for 10 s by superfusion with an extracellular solution titrated to pH 6 containing (in mM) 140 NaCl, 5 KCl, 2 CaCl_2_, 1 MgCl_2_, 10 2-N-morpholinoethanesulfonic acid (MES) (instead of HEPES), and 10 glucose using the multi-barrel perfusion pipette. All experiments were done under control conditions and after adding either 0.5 µM SP (Tocris Bioscience, Toc-1156-M005, Bristol, GB) or 0.5 µM CGRP (Tocris Bioscience, Toc-1161-C100, Bristol, GB) to the superfusate in the multi-barrel perfusion pipette, respectively [54]. Both substances were dissolved in Aqua Dest., stored at − 20 °C and diluted in the extracellular solution just before experiments. The dose of 0.5 µM of SP and CGRP was chosen as described by others [54]. This concentration of both substances did not elicit action potentials or altered inward currents during current clamp or voltage clamp protocols, respectively (see below).

### Current injection

In another set of experiments, action potential generation was induced by rectangular current-pulse injections as follows: A 5-ms pre-pulse, followed by a 600-ms lasting pulse with an inter-pulse delay of 100 ms, was delivered in three consecutive trains of increased intensity (40–400 pA, 400–4000 pA, 4000–12,000 pA) in 10 consecutive steps (5.16 s, each). We categorized DRG neurons as “tonic” or “phasic” as described previously [54]: Neurons generating 5 or more APs were defined as “tonic.” In contrast, neurons generating one to four APs were defined as “phasic.”

### Voltage clamp protocols—Effects of SP and CGRP

Each neuron that was stimulated by protons to generate APs in current clamp mode underwent also a voltage clamp protocol to investigate the acid-induced currents upon proton stimulation with and without SP or CGRP.

Sustained currents were evoked by 10-s lasting acidic superfusion (pH 6) with or without addition of SP or CGRP (0.5 µM) once every 40 s three times in a row using the multi-barrel perfusion pipette. The investigated cell was exposed either to protons or to acidified solution containing SP or CGRP (0.5 µM) or SP or CGRP alone (0.5 µM). Acid-induced currents showed a transient and a sustained component. Sustained currents are likely to be TRPV1-mediated because the TRPV1 channel inhibitor capsazepine was able to block this current, as demonstrated earlier [16]. In order to compare the acid-sensitive sustained currents in different cells, we measured current amplitudes that were evoked upon proton stimulation (e.g., baseline currents were subtracted). For these protocols, the same solutions were used as outlined above.

### Data analysis

**Fig. 1:**
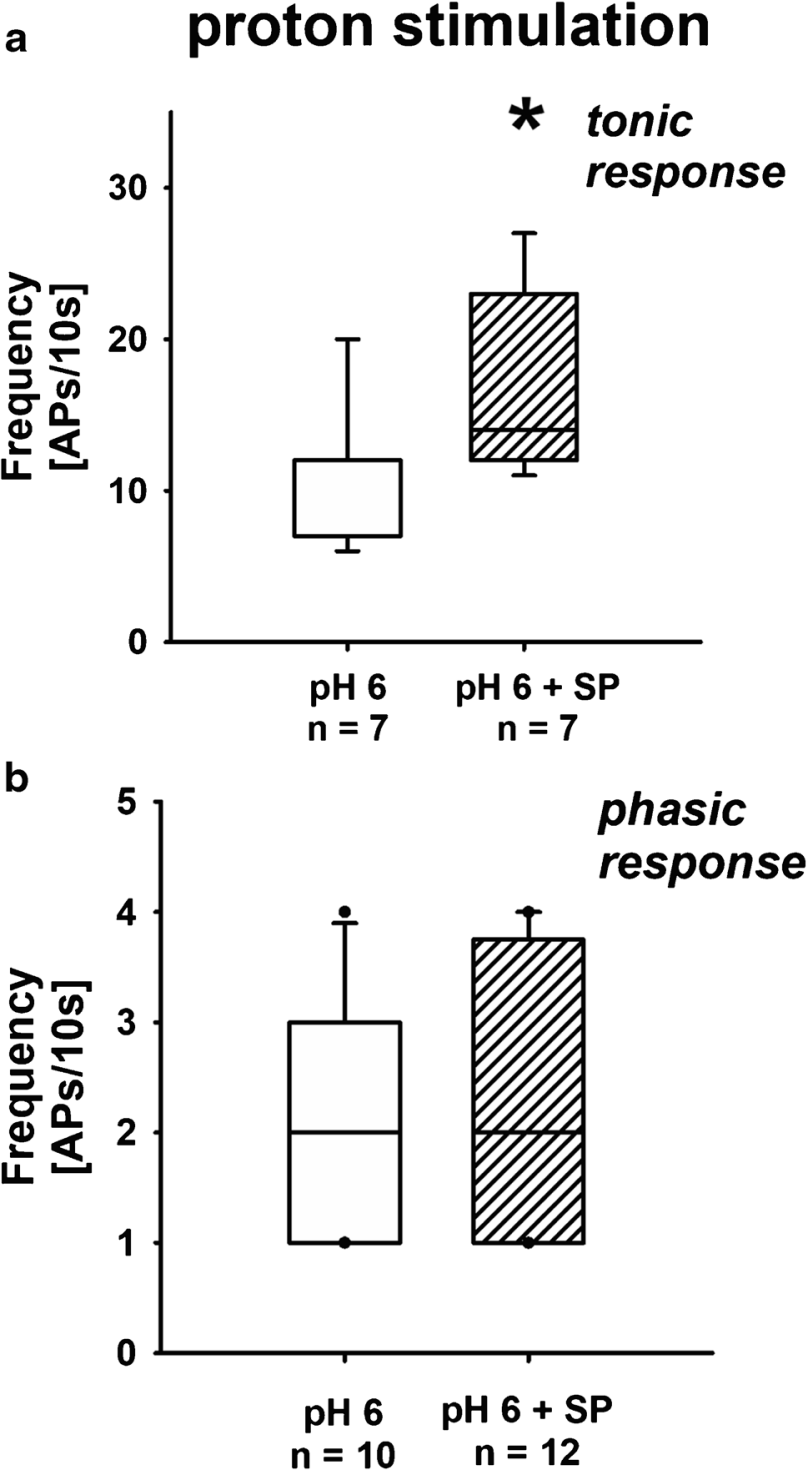
AP generation due to acid stimulation: effect of SP. **a** When SP was added, administration of an acidic extracellular solution (pH 6) increased action potential production significantly as compared to acidic solution alone. Data presented as box plots and whiskers showing median values, 1st and 3rd quartiles, and 5th and 95th percentiles (outlayers are presented as dots) (pH 6 vs. pH 6 + SP, KSt failed, Mann–Whitney rank sum test, *P = 0.018). **b** No effect of SP was seen in phasic renal neurons. Data presented as box plots and whiskers showing median values, 1st and 3rd quartiles, and 5th and 95th percentiles (outlayers are presented as dots) (pH 6 vs pH 6 + SP, KSt failed, Mann–Whitney rank sum test, *P* = 0.843)

**Fig. 2:**
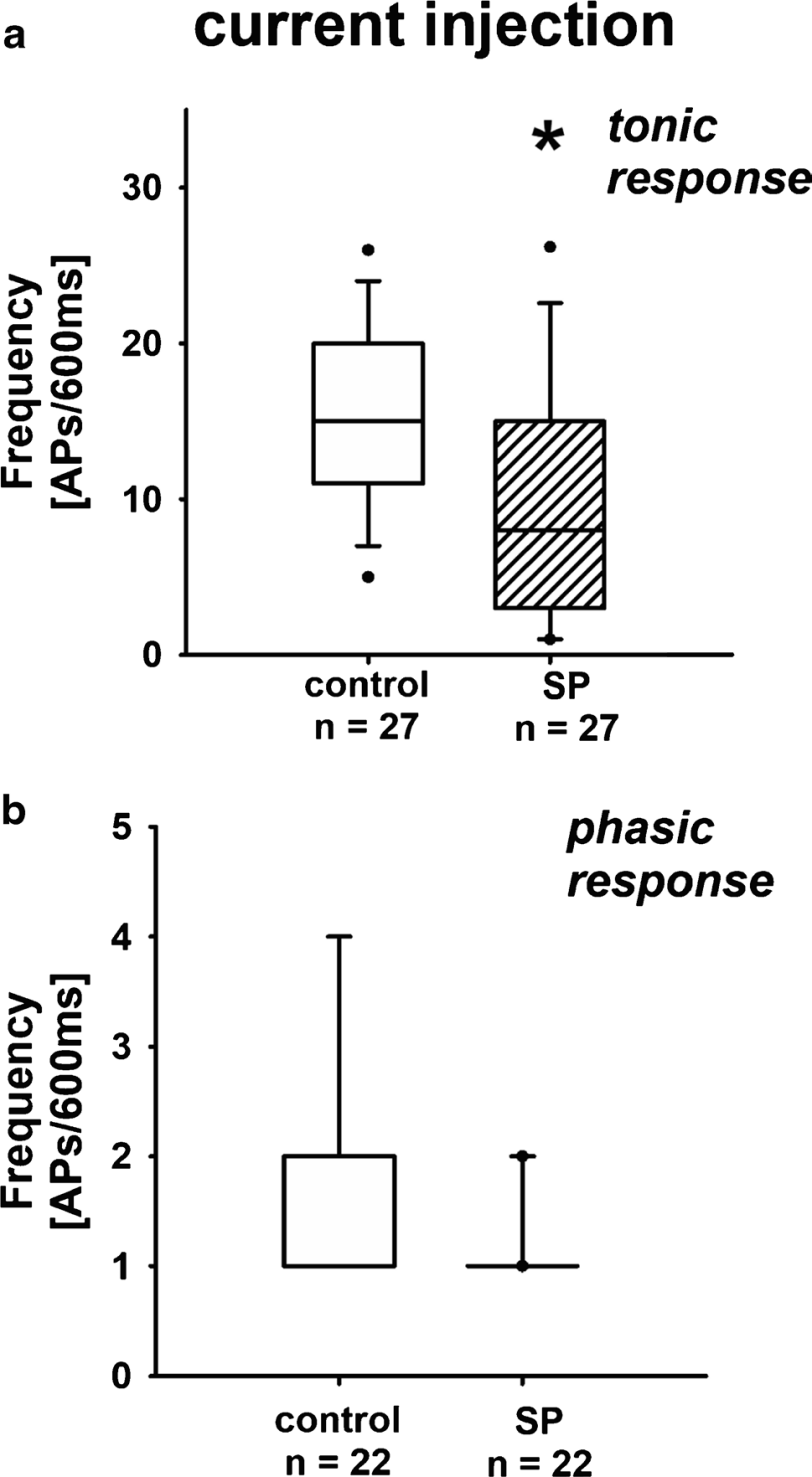
Frequency of action potentials due to current injection in tonic and phasic neurons: effect of SP. **a** Exposure of tonic renal neurons to SP significantly decreased the action potential generation due to current injection. Data presented as box plots and whiskers showing median values, 1st and 3rd quartiles, and 5th and 95th percentiles (outlayers are presented as dots) (control vs. SP, KSt failed, Wilcoxon signed rank test, *P < 0.0001). **b** SP had no effect on phasic neurons in this setting. Data presented as box plots and whiskers showing median values, 1st and 3rd quartiles, and 5th and 95th percentiles (outlayers are presented as dots) (controls vs. SP, KSt failed, Wilcoxon signed rank test, *P* = 0.063)

**Fig. 3:**
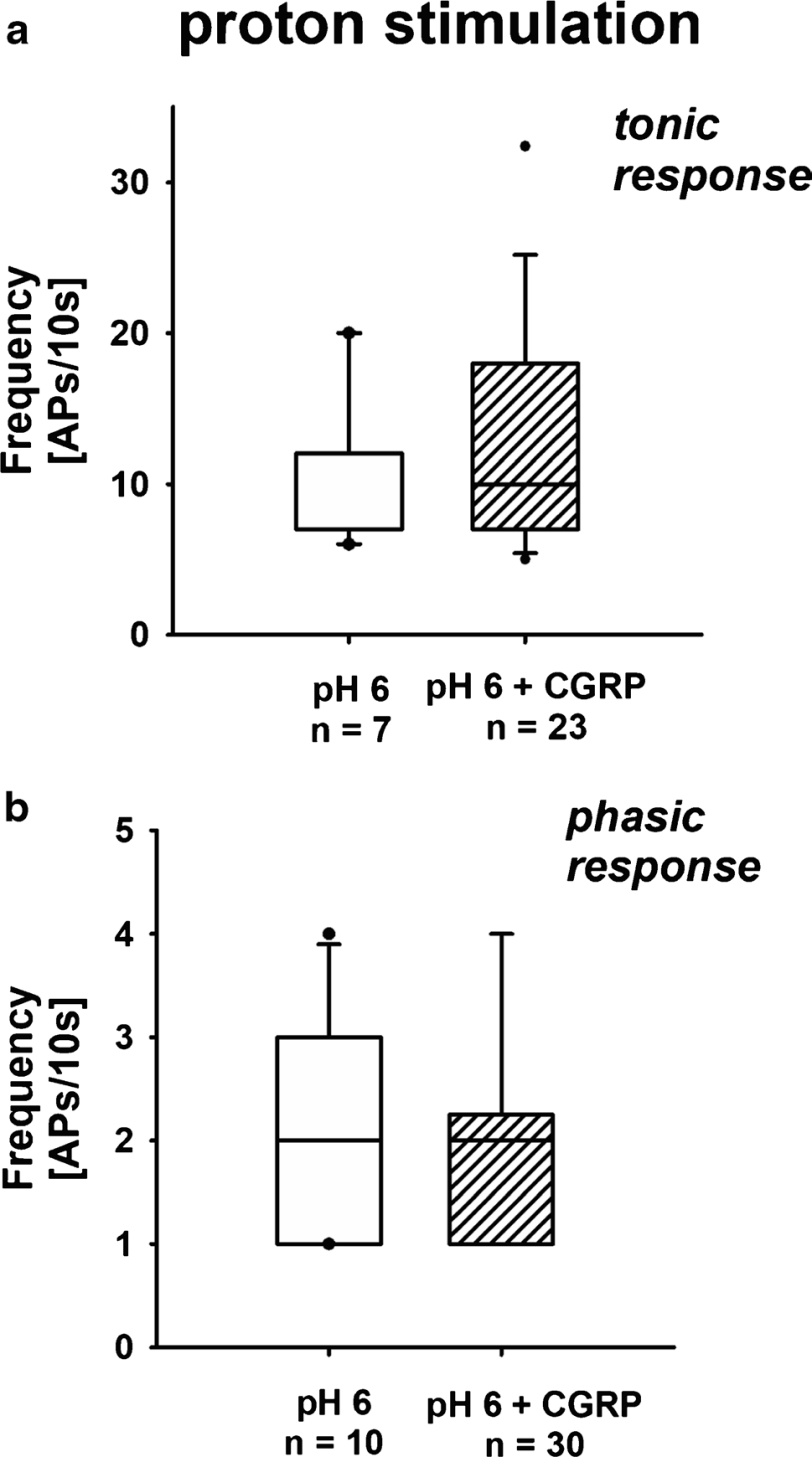
AP generation due to acid stimulation: effect of CGRP. **a** Exposure of renal tonic neurons to CGRP did not affect action potential generation due to acidic stimulation. Data presented as box plots and whiskers showing median values, 1st and 3rd quartiles, and 5th and 95th percentiles (outlayers are presented as dots) (control vs. CGRP, KSt failed, Mann–Whitney rank sum test, *P* = 0.327). **b** Phasic renal neurons also remained unaffected by CGRP exposure. Data presented as box plots and whiskers showing median values, 1st and 3rd quartiles, and 5th and 95th percentiles (outlayers are presented as dots) (control vs. CGRP, KSt failed, Mann–Whitney rank sum test, *P* = 0.754)

**Fig. 4:**
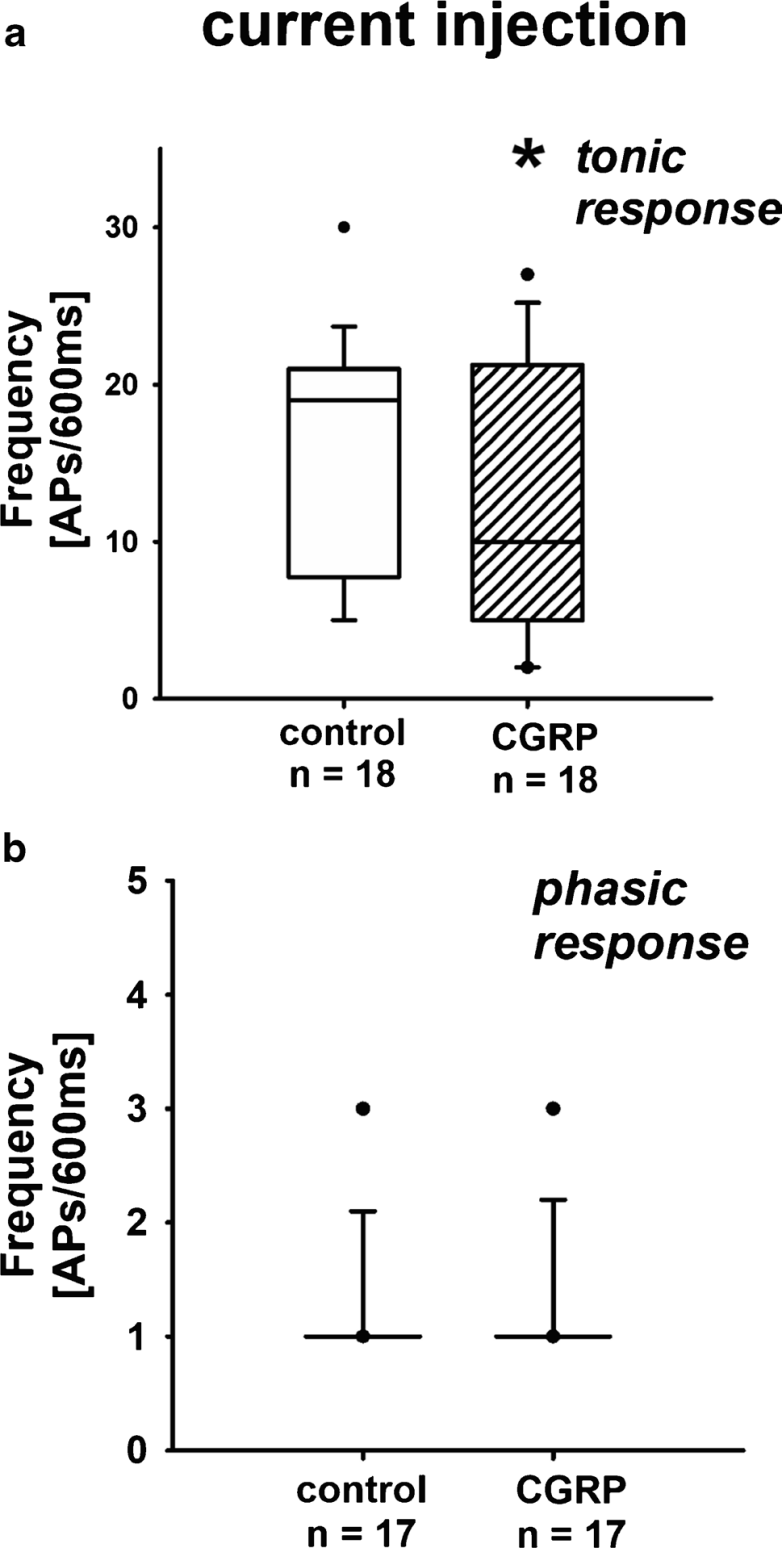
AP generation due to current injection: effect of CGRP. **a** Exposure of tonic renal neurons to CGRP significantly decreased the action potential generation due to current injection. Data presented as box plots and whiskers showing median values, 1st and 3rd quartiles, and 5th and 95th percentiles (outlayers are presented as dots) (CGRP vs. control, KSt failed, Wilcoxon signed rank test, *P = 0.039). **b** CGRP had no effect on phasic neurons in this setting. Data presented as box plots and whiskers showing median values, 1st and 3rd quartiles, and 5th and 95th percentiles (outlayers are presented as dots) (CGRP vs. control, KSt failed, Wilcoxon signed rank test, *P* = 1)

**Fig. 5:**
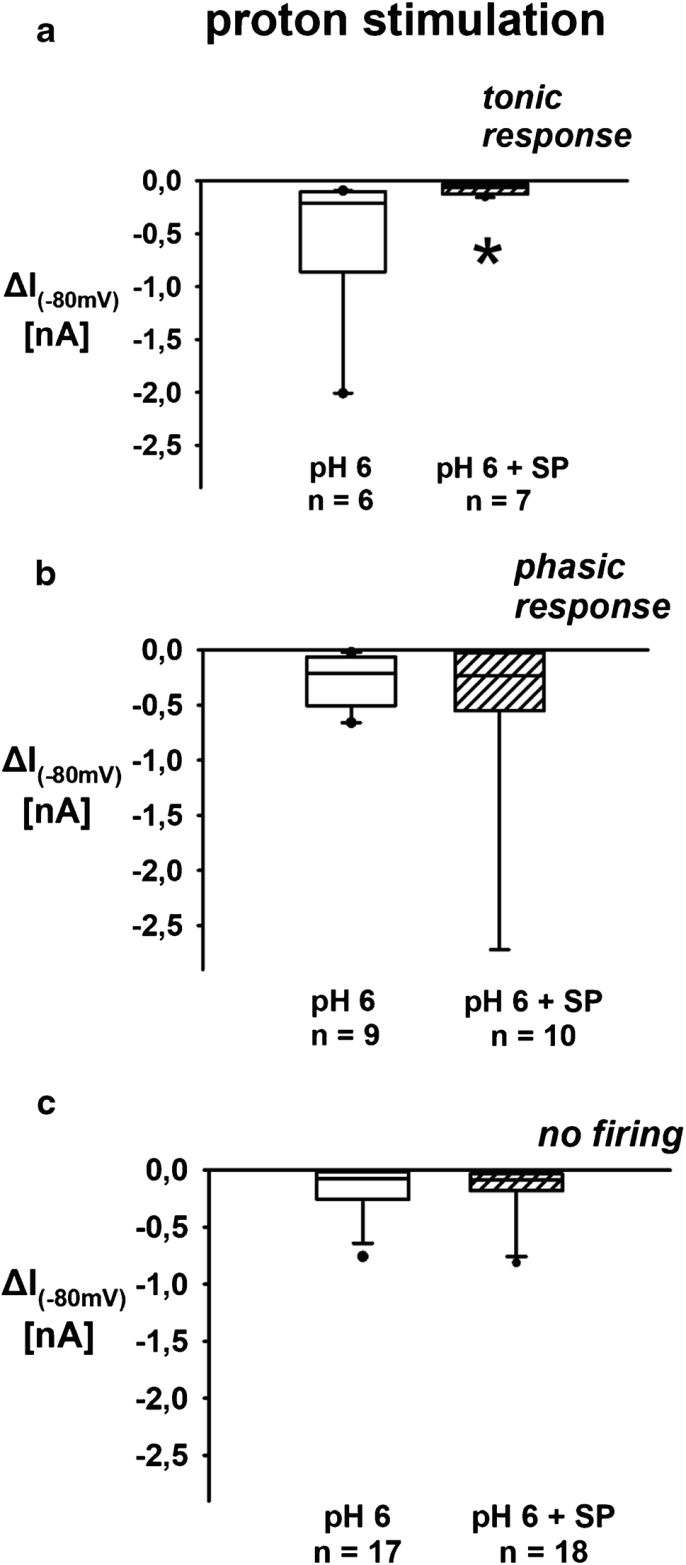
Proton-induced sustained inward currents of renal neurons: effect of SP. **a** SP significantly decreased sustained proton-induced inward currents in tonic renal neurons. Data presented as box plots and whiskers showing median values, 1st and 3rd quartiles, and 5th and 95th percentiles (outlayers are presented as dots) (pH 6 vs. pH 6 + SP, KSt failed, Mann–Whitney rank sum test, *P = 0.035). **b** SP did not affect sustained proton-induced inward currents in renal phasic neurons. Data presented as box plots and whiskers showing median values, 1st and 3rd quartiles, and 5th and 95th percentiles (outlayers are presented as dots) (pH 6 vs. pH 6 + SP, KSt failed, Mann–Whitney rank sum test, *P* = 0.967). **c** Sustained proton-induced inward current in renal neurons with no firing response to acidic stimulation was not altered under SP. Data presented as box plots and whiskers showing median values, 1st and 3rd quartiles, and 5th and 95th percentiles (outlayers are presented as dots) (pH 6 vs. pH 6 + SP, KSt failed, Mann–Whitney rank sum test, *P* = 0.947)

**Fig. 6:**
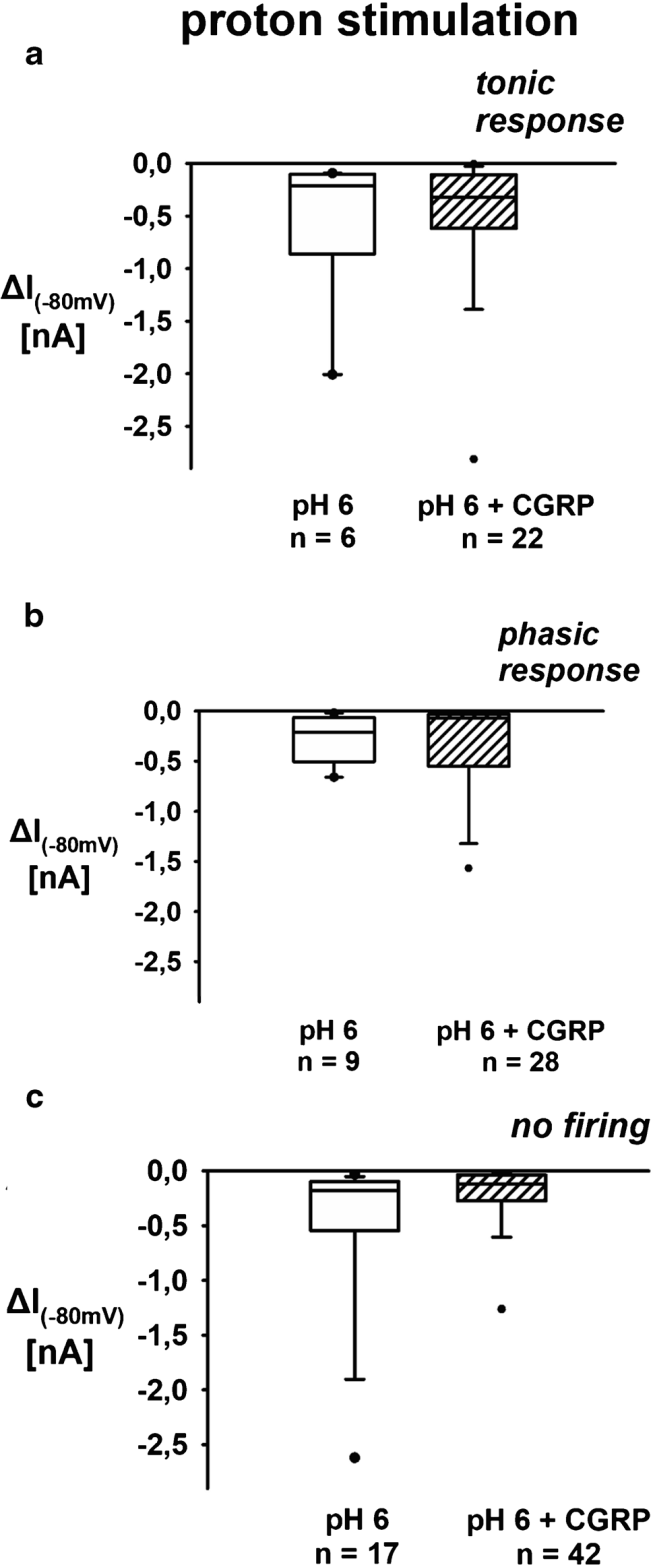
Proton-induced sustained inward currents of renal neurons: effect of CGRP. **a** CGRP does not affect proton-induced sustained inward currents in tonic renal neurons. Data presented as box plots and whiskers showing median values, 1st and 3rd quartiles, and 5th and 95th percentiles (outlayers are presented as dots) (pH 6 vs. pH 6 + CGRP, KSt failed, Mann–Whitney rank sum test, *P* = 0.884). **b** CGRP does not affect proton-induced sustained inward currents in phasic renal neurons. Data presented as box plots and whiskers showing median values, 1st and 3rd quartiles, and 5th and 95th percentiles (outlayers are presented as dots) (pH 6 vs. pH 6 + CGRP, KSt failed, Mann–Whitney rank sum test, *P* = 0.583). **c** CGRP does not affect proton-induced sustained inward currents in non-firing renal neurons. Data presented as box plots and whiskers showing median values, 1st and 3rd quartiles, and 5th and 95th percentiles (outlayers are presented as dots) (pH 6 vs. pH 6 + CGRP, KSt failed, Mann–Whitney rank sum test, *P* = 0.738)

Normality testing was performed by the Kolmogorov–Smirnov test. Normally distributed data were tested using a *t*-test. Otherwise, non-parametric testing (Mann–Whitney rank sum test) was performed. For comparison of more than two normally distributed groups, one-way ANOVA with a Student–Newman–Keuls post hoc test was executed. Otherwise, Kruskal–Wallis one-way ANOVA of ranks was used with Dunn’s post hoc method. Statistical significance was defined as *P* < 0.05. The *z*-test was used to test for significant differences in the frequency distribution of firing characteristics of the DRG neurons (tonic vs. phasic for neurons with renal and non-renal axons). These data are presented as pie graphs. SigmaPlot 14 and SigmaStat 3.5 (Systat Software, Erkrath, Germany) were used for statistical analysis and graphical display. Data are given as group means ± SD in the text and tables, and presented as box plots and whiskers in Figs. 1, 2, 3, 4, 5, and 6 showing median values, 1st and 3rd quartiles, and 5th and 95th percentiles (outliers are presented as dots).

## Results

Cultured neurons could be distinguished from fibroblasts and other cells due to their typical size and soma. Approximately 25% of these neurons were brightly labelled with DiI indicating that these neurons received projections from the kidney [13, 23]. The largest group of DRG neurons under investigation was represented by medium-capacitance and medium-sized cells, as previously described [16].

### Stimulation with acidic solutions

When the neurons were stimulated with acidic solution (pH 6) that was directly applied via the multi-barrel perfusion pipette, the frequency of the acid-induced action potentials increased significantly in renal tonic neurons if exposed to SP (Fig. 1; supplementary material: Section A). No such effect was observed with CGRP exposure (Fig. 3; supplementary material: Section C).

However, when current injection was applied, the firing frequency of renal cells with tonic response pattern was decreased if neurons were exposed to SP (Fig. 2; supplementary material: Section B). Under these circumstances, exposure to CGRP similarly reduced the number of action potentials in tonic neurons significantly (Fig. 4; supplementary material: Section D). Neither exposure to SP nor to CGRP affected neurons with phasic response pattern (Figs. 1, 2, 3, and 4).

### Voltage clamp recordings

In voltage clamp recordings, the addition of SP significantly reduced the acid-evoked sustained TRPV1-mediated currents (Fig. 5; supplementary material: Section E). This effect again was only observed in renal tonic but not in renal phasic neurons (− 518 ± 743 pA due to pH 6 superfusion vs. − 82 ± 50 pA due to pH 6 and SP superfusion). Addition of CGRP had no effects on inward currents (Fig. 6; supplementary material: Section F).

If neurons were not able to generate APs due to stimulation with protons in the current clamp mode, they were subsumed neither to the phasic nor to the tonic neuron group. Therefore, there are three groups of cells displayed in Figs. 5 and 6.

Superfusion with SP or CGRP alone did not induce inward currents in voltage clamp recordings. Furthermore, neither SP nor CGRP alone was able to induce APs in current clamp recordings.

**Fig. 7:**
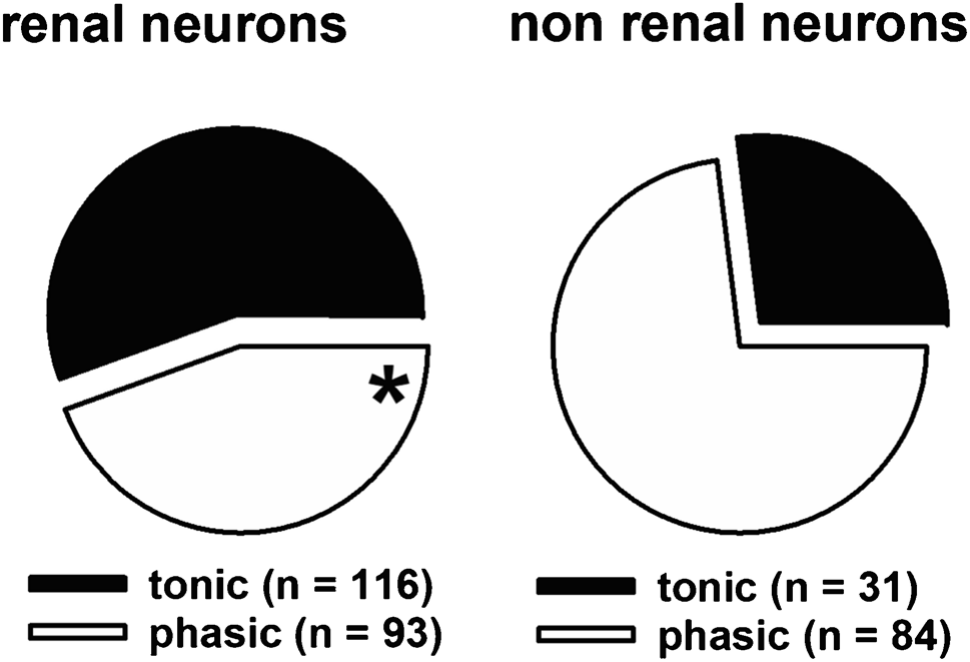
Ratio of tonic to phasic neurons in samples of neurons with axons from the kidney (*n* = 209; left pie chart) and from non-renal sites (*n* = 115; right pie chart). The renal sample is characterized by a higher number neurons with a tonic response due to current injection (56% tonic neurons in a renal group vs. 26% of tonic neurons in non-renal sample (*z*-test, *P < 0.001)

Renal neurons proved again to comprise a significant higher amount of neurons with a tonic response pattern upon stimulation as compared to neurons with projections from non-renal sites (Fig. 7).

### Electrophysiological properties of investigated neurons

Neurons were further investigated to characterize their electrophysiological properties, i.e., capacity, membrane resistance, and resting potential. These data were recorded at the very beginning of the experimental protocols of each cell. Thus, no changes of these data due to the effect of SP or CGRP were recorded.

As in previous experiments of ours [13, 23], in any experimental setup, tonic neurons exhibited a higher action potential frequency, a higher threshold, a broader action potential, and a higher peak potential as compared to phasic neurons (Table 1: experiments with SP, Table 2: experiments with CGRP).

**Table 1:**
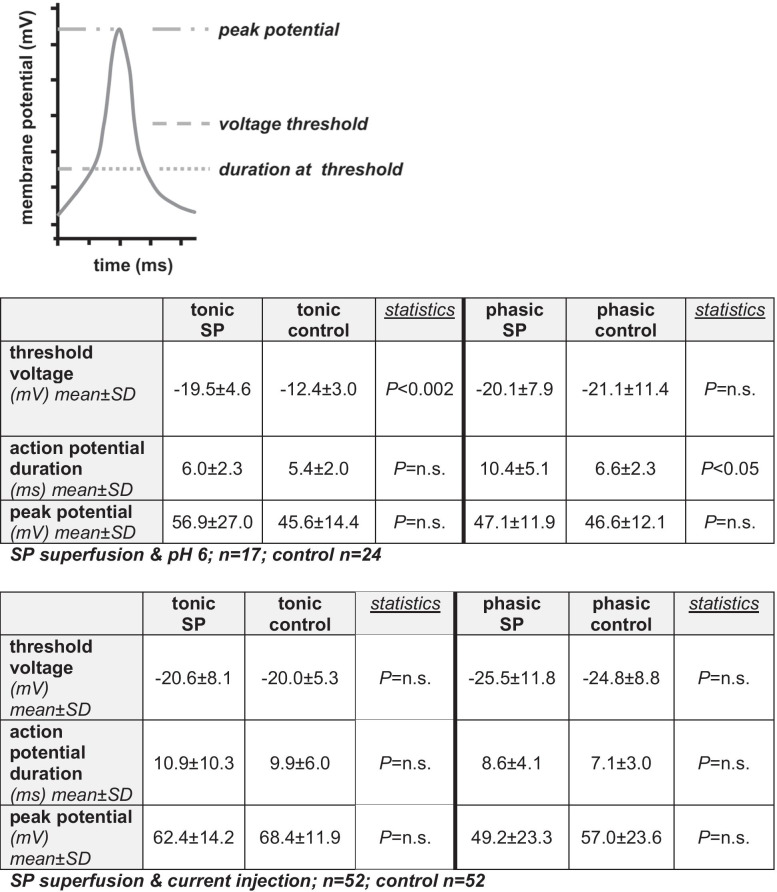
Electrophysiological characteristics of renal neurons (dorsal root ganglion (DRG) neurons with afferent axons from the kidneys)

**Table 2:** Electrophysiological characteristics of renal neurons (DRG neurons with afferent axons from the kidneys)

	Tonic CGRP	Tonic control	Statistics		Phasic CGRP	Phasic control		Statistics
CGRP superfusion and pH 6; *n* = 24; control *n* = 24								
Threshold voltage (mv) mean ± SD	− 15.8 ± 4.2	− 12.4 ± 3.0	*P* < 0.001		− 17.9 ± 8.9	− 20.2 ± 11.2		*P* = n.s
Action potential duration (ms) mean ± SD	6.1 ± 3.1	5.4 ± 2.4	*P* = n.s		10.8 ± 5.9	6.6 ± 2.2		*P* < 0.05
Peak potential (mv) mean ± SD	48.0 ± 11	45.6 ± 14.4	*P* = n.s		44.2 ± 16.6	46.6 ± 12.3		*P* = n.s
CGRP superfusion and current injection; *n* = 41; control *n* = 41								
Threshold voltage (mV) mean ± SD	− 11.1 ± 6.4	− 12.9 ± 5.9		*P* < 0.05	− 12.8 ± 11.2		− 17.7 ± 13.1	*P* < 0.05
Action potential duration (ms) mean ± SD	5.5 ± 3.6	5.5 ± 3.5		*P* = n.s	4.4 ± 2.0		5.8 ± 4.2	*P* = n.s
Peak potential (mV) mean ± SD	51.5 ± 9.7	58.2 ± 10.9		*P* < 0.001	35.4 ± 23.7		52.4 ± 21.1	*P* < 0.001

### Neurons with projections from non-renal sites

In Table 3 (SP) and Table 4 (CGRP), the data of neurons from non-renal sites are shown. These tables also present AP production upon respective stimulation. In general, responses were far less consistent than in neurons having renal projections.

**Table 3:** Electrophysiological characteristics of non-renal neurons (dorsal root ganglion neurons with afferent axons from other sites than kidneys)

	Tonic SP	Tonic control	Statistics	Phasic SP	Phasic control	Statistics	
SP superfusion and current injection; *n* = 23; control *n* = 23							
Frequency of action potentials (APs/600 ms) mean ± SD	8.4 ± 8.7	14.1 ± 6.3	*P* < 0.05	1.1 ± 0.3	1.8 ± 1.2		*P* = n.s
Threshold voltage (mV) mean ± SD	− 23.4 ± 9.4	− 25.33 ± 6.4	*P* = n.s	23.1 ± 7.8	− 29 ± 11.7		*P* = n.s
Action potential duration (ms) mean ± SD	10.4 ± 5.9	10.4 ± 6.4	*P* = n.s	6.3 ± 3.1	6.5 ± 2.7		*P* = n.s
Peak potential (mV) mean ± SD	55.9 ± 19.2	66.3 ± 14.4	*P* < 0.05	39.2 ± 19.4	49.6 ± 20.1		*P* < 0.05
SP superfusion and pH 6; *n* = 22 (non-firing neurons); control *n* = 57 (7 firing neurons, 12%)							
Frequency of action potentials (APs/10 s) mean ± SD	No APs	5	*P* = n.s	No APs	2.2 ± 1.5		*P* = n.s
Threshold voltage (mV) mean ± SD	No APs	− 23.5	*P* = n.s	No APs	− 13.3 ± 3.8		*P* = n.s
Action potential duration (ms) mean ± SD	No APs	5.9	*P* = n.s	No APs	7.7 ± 3.4		*P* = n.s
Peak potential (mV) mean ± SD	No APs	28	*P* = n.s	No APs	46 ± 12.9		*P* = n.s

Non-renal neurons performed only once tonic firing when stimulated with pH 6. All other neurons showed phasic or no firing (0–4 action potentials upon 10 s of stimulation). No action potentials were generated in non-renal neurons when SP 0.5 µM was added to acidic solution (pH 6)

**Table 4:** Electrophysiological characteristics of non-renal neurons (dorsal root ganglion neurons with afferent axons from other sites than the kidneys)

	Tonic CGRP	Tonic control	Statistics	Phasic CGRP	Phasic control	Statistics
CGRP superfusion and current injection; *n*=29; control *n*=29						
Frequency of action potentials (APs/600ms) mean±SD	4.8±5.6	14.8±5.6	*P*=n.s.	1.1±0.5	1±0	*P*=n.s.
Threshold voltage (mV) mean±SD	−13.8±9	−15±8	*P*=n.s.	−20.6±8.7	−23.6±6.3	*P*<0.05
Action potential duration (ms) mean±SD	5.6±4.2	5.9±3.9	*P*=n.s.	3±1.9	3.6±3.2	*P*=n.s.
Peak potential (mV) mean±SD	40.5±18	49.8±20	*P*=n.s.	33±20	38.8±19	*P*<0.001
CGRP superfusion and pH 6; *n*= 95 (4 firing neurons, 4%); control *n*=57 (7 firing neurons, 12%)						
Frequency of action potentials (APs/10s) mean±SD	8	5	*P*=n.s.	3±1	2.2±1.5	*P*=n.s.
Threshold voltage (mV) mean±SD	−27	−23.5	*P*=n.s.	−27.7±19.4	−13.3±3.8	*P*=n.s.
Action potential duration (ms) mean±SD	4.2	5.9	*P*=n.s.	5.2±2.0	7.7±3.4	*P*=n.s.
Peak potential (mV) mean±SD	62	28	*P*=n.s.	51±17.3	46±12.9	*P*=n.s.

12% of non-renal neurons fired action potentials when stimulated with protons (7 out of 57 investigated neurons). Only one neuron showed tonic firing upon 10 s of stimulation. 4% of neurons (4 out of 95 investigated neurons) generated action potentials when CGRP 0.5 µM was added to acidic solution. Only one neuron showed tonic firing upon 10 s of stimulation (no significant difference, *z*-test)

## Discussion

In this study, we tested the hypothesis that substance P (SP) released from afferent renal nerves inhibits the action potential production in neurons with renal afferent projections. However, in contrast to our assumption, SP led to an increased frequency of action potentials in tonic renal neurons, when the generation of action potentials was triggered by the stimulation of TRPV1 receptors via protons (pH 6). Under these conditions, on the other hand, the inward currents of these renal neurons were reduced as could be shown by voltage clamp experiments. If we now triggered action potentials of tonic renal neurons via a very general stimulus such as current injections, the presence of substance P resulted in a reduction of the action potential generation compared to control conditions, which we had originally expected based on our data from recently published studies [48, 51]. During current injections, the presence of CGRP also reduced the frequency of action potential formation compared to controls, which had no effect when TRPV1 receptors were stimulated via protons.

Comparable results could not be obtained in phasic neurons [16, 23].

Again, it was found that tonic neurons of the kidney far outweighed tonic neurons in non-renal samples [16]. Although the general classification into tonic and phasic neurons is also possible for non-renal samples, the tonic neurons appeared to be less responsive to our experimental interventions. However, due to the small number of tonic neurons in the non-renal group, the physiological meaning of the finding is at least questionable.

### SP—Importance for TRPV1 sensitization

Our finding that stimulation of TRPV1 receptors with protons led to an increased generation of action potentials when the cells were exposed to a solution containing substance P is in accordance with publications on SP increasing the sensitivity of TRPV1 receptors [20, 33]. The SP receptor tachykinin-1, neurokinin-1 (NK1), was described to be densely expressed in the superficial dorsal horn [55] and in dorsal root ganglion neurons together with TRPV1 receptors [32]. Further reports suggest that NK1 receptors, which represent the binding site of SP, are not only co-expressed but functionally linked to TRPV1 receptors in primary peptidergic afferent neurons in the dorsal root ganglia [35, 60]. SP appeared to induce its effects less via PKC inhibition [33], which is known to lower the activation threshold of TRPV1 [41], than via a polyubiquitination of TRPV1, a post-translational modification shared by several cellular pathways that include endosomal recycling and proteosomal degradation [38].

TRPV1 is a member of the tetrameric cation channel superfamily. The channel complex has been extensively studied [8, 34] and consists of four subunits arranged symmetrically around a central ion permeation pore [63]. There are numerous acidic residues in the outer pore region as well as the peripheral extracellular loops that are seen to serve as potential protonation sites for channel gating: Specific amino acid mutations at these sites were found to have dramatic effects on H^+^-induced channel activation [27].

In this context, it should be noted that receptors on tonic neurons that can be stimulated via protons, such as TRPV1 [4, 39] or ASIC [44], exhibit mechanosensory properties. We could show a substance P–dependent sympatho-inhibitory control circuit via afferent nerves even under healthy conditions [14]. Mice lacking NK1 receptors have an increased blood pressure [40]. The authors, who examined these mice, assumed a neurogenic mechanism rather than a change in vascular reactivity of these mice. These findings suggest that SP in the kidney might affect neural control of regional blood flow, and can influence the activity of the sympathetic nervous system to the kidney by influencing mechanosensitive mechanisms of afferent nerve pathways. The fact that during stimulation of TRPV1 receptor by protons the presence of substance P reduced inward currents across the membrane in general (not only linked to action potential generation) is in accordance with such a regulatory mechanism: Neuronally released SP could, after release, hinder its own further secretion in order to regulate the influence on sympathetic nerve activity via afferent nerve pathways in the sense of a negative feedback.

### Stimulation of renal afferent neurons?

SP led according to some reports to a sensitized action potential formation on current injections. This is attributed to an effect of SP on Nav 1.8 [7, 61]. These channels with a high expression in renal neurons [23] likely play a prominent role in repetitive firing characterizing tonic renal neurons in that they uniquely showed slow activation and a fast recovery at depolarized membrane potentials after inactivation [19, 47, 53].

However, in the experiments with SP mentioned above, the peptide was added in a concentration that already led to quiescent current changes [7]. This was not the case in our experiments, where we added SP (or CGRP) to the superfusion medium and thus to the patch clamp bath solution. Hence, it is not surprising that the responses to current injections in our experiments were also different from the results mentioned above in that there was significantly less action potential formation in tonic renal neurons in the presence of SP or CGRP as compared to control conditions. In previous experiments of ours, renal neurons were incubated with CXCL1 for 1 day, and there were no quiescent current changes. In these experiments, the tonic neurons were still able to achieve maximum action potential formation during current injection as under control conditions [13]. However, the number of tonic neurons decreased significantly, so that it must be assumed that the maximum achievable total activity of an afferent nerve fiber bundle consisting of many single filaments is also reduced in vivo.

Although the mechanisms behind our own observations are not yet clarified in detail, they are likely to be relevant under in vivo conditions. It is not to be expected that vasoactive and/or proinflammatory substances are always produced in the kidney in concentrations that lead to overthreshold electrophysiological responses [51, 57]. Our own recently published reports have also clearly suggested an impaired activity of afferent renal nerve pathways that under these circumstances have most likely lost sympathetic inhibitory potential [48, 51].

Our findings in this paper and previous publications [14, 49, 51] as well as those of other authors [28, 29] argue for a sympatho-inhibitory effect of renal afferents under normal conditions and a loss of sympatho-inhibition in pathological situations. However, we are well aware that there are also reports [50, 59, 62] suggesting sympatho-excitatory influences of afferent renal nerves. Probably, we must assume that both populations exist, without being able to say so far exactly when which population exerts which influence on central nervous sympathetic production under which stimulation conditions. The contradictions still existing here at present may also have to do with the fact that between afferent nerves and their influence on central nervous outflow, there is still the central nervous processing of afferent inputs, which could also exert different influences on sympathetic activity in different pathological situations.

In summary, at least three situations can be distinguished for afferent renal nerve units: increased stimulation of tonic neurons via specific mechanisms (e.g., TRPV1 receptor mediated) under acidic conditions, reduced excitability of these units due to electrical stimulation, and the putative decrease of tonic nerve fibers in favor of phasic units when interacting with cytokines. Further experiments in vivo and in vitro will have to clarify more precisely the exact mechanisms of SP influence. More than that, SP could conceivably affect interaction between neurons and immune cells via NK1 receptors or via intracellular pathways using second messengers, controlling the expression of cytokines and modulating TRPV1 receptor or NK1 receptor activity.

## Supplementary Information

Below is the link to the electronic supplementary material.

Supplementary file1 (DOCX 1103 KB)
