# Supplementary material for: Neurogenic substance P—influences on action potential production in afferent neurons of the kidney?
Source: Pflugers Arch. 2021 Mar 30;473(4):633–46. doi: 10.1007/s00424-021-02552-z (PMC8049925; doi:10.1007/s00424-021-02552-z)
Supplement: Supplementary file 1 — Supplementary file1 (DOCX 1103 KB) [file 424_2021_2552_MOESM1_ESM.docx]

***Supplementary Material***

**Neurogenic Substance P - influences on action potential production in afferent neurons of the kidney?**

*Revision 2 PAEJ-D-20-00375*

Kristina Rodionova^1^, Karl F. Hilgers^1^, Peter Linz^4^, Johannes Schätzl^1^, Giulia Raschke^1^, Christian Ott^1,2^, Roland E. Schmieder^1,2^, Mario Schiffer^1^, Kerstin Amman^1^, Roland Veelken^1,2^, Tilmann Ditting^1,2^

^1^ Department of Internal Medicine 4 - Nephrology and Hypertension,

Friedrich-Alexander University Erlangen, Germany

^2^ Department of Internal Medicine 4 – Nephrology and Hypertension,

Paracelsus Private Medical School Nuremberg, Germany

^3^ Department of Nephropathology, Friedrich-Alexander University Erlangen, Germany

^4^ Department of Radiology, Friedrich-Alexander University Erlangen, Germany

**Section 1: Representative Recordings of Action Potentials during Stimulation with Acidic Solutions and Substance P**

**Fig. S1A:** Proton induced (pH 6) action potentials in tonic renal neurons without (left upper panel) and with added SP (right upper panel). The lower panels display the first 1.2 Seconds of stimulation. Individual action potentials are visible (left lower panel without and right lower panel with added SP).

|  |  |
| --- | --- |
|  |  |

**Fig. S1B:** Proton induced (pH 6) action potentials in phasic renal neurons without (left panel) and with added SP (right panel). The lower panels display the first 1.2 seconds of stimulation. Individual action potentials are visible. The lower panels display the first 1.2 seconds of stimulation. Individual action potentials are visible (left lower panel without and right lower panel with added SP).

|  |  |
| --- | --- |
|  |  |

**Section 2: Representative Recordings of Action Potentials due to Current Injection and Substance P**

**Fig. S2A:** Current induced action potentials in tonic renal neurons without (left panel) and with added SP (right panel).

|  |  |
| --- | --- |

**Fig. S2B:** Current induced action potentials in phasic renal neurons without (left panel) and with added SP (right panel)

|  |  |
| --- | --- |

**Section 3: Representative Recordings of Action Potentials during Stimulation with Acidic Solutions and CGRP**

**Fig. S3A:** Proton induced (pH 6) action potentials in tonic renal neurons without (left panel) and with added CGRP (right panel). The lower panels display the first 1.2 Seconds of stimulation. Individual action potentials are visible (left lower panel without and right lower panel with added CGRP).

|  |  |
| --- | --- |
|  |  |

**Fig. S3B:** Proton induced (pH 6) action potentials in phasic renal neurons without (left panel) and with added CGRP (right panel). The lower panels display the first 1.2 Seconds of stimulation. Individual action potentials are visible (left lower panel without and right lower panel with added CGRP).

|  |  |
| --- | --- |
|  |  |

**Section 4: Representative Recordings of Action Potentials due to Current Injection and CGRP**

**Fig. S4A:** Current induced action potentials in tonic renal neurons without (left panel) and with added CGRP (right panel)

|  |  |
| --- | --- |

**Fig. S4B:** Current induced action potentials in phasic renal neurons without (left panel) and with added CGRP (right panel)

|  |  |
| --- | --- |

**Section 5: Representative Voltage Clamp Recordings of Sustained Inward Current during Stimulation with Acidic Solution and Substance P**

**Fig. S5A:** Proton induced sustained inward currents (pH 6) in tonic renal neurons without (left panel) and with added SP (right panel)

|  |  |
| --- | --- |

**Fig. S5B:** Proton induced sustained inward currents (pH 6) in phasic renal neurons without (left panel) and with added SP (right panel)

|  |  |
| --- | --- |

**Fig. S5C:** Proton induced (pH 6) sustained inward currents in renal neurons with no firing response to acidic stimulation: proton stimulation (left panel) and with added SP (right panel)

|  |  |
| --- | --- |

**Section 6: Representative Voltage Clamp Recordings of Sustained Inward Current during Stimulation with Acidic Solution and CGRP**

**Fig. S6A:** Proton induced sustained inward currents (pH 6) in tonic renal neurons without (left panel) and with added CGRP (right panel)

|  |  |
| --- | --- |

**Fig. S6B:** Proton induced sustained inward currents (pH 6) in phasic renal neurons without (left panel) and with added CGRP (right panel)

|  |  |
| --- | --- |

**Fig. S6C:** Proton induced (pH 6) sustained inward currents in renal neurons with no firing response to acidic stimulation: proton stimulation (left panel) and with added CGRP (right panel)

|  |  |
| --- | --- |
